# How could fit between polychronicity and multitasking shape employees' self-leadership? The moderating role of AI-empowered task processing

**DOI:** 10.3389/frai.2025.1451944

**Published:** 2025-01-28

**Authors:** Zhirui Zhou, Shuting Xiang, Qingxin Xie

**Affiliations:** ^1^School of International Business, Southwestern University of Finance and Economics, Chengdu, China; ^2^College of Management, Sichuan Agricultural University, Chengdu, China

**Keywords:** polychronicity, multitasking, polychronicity-multitasking fit, artificial intelligence empowerment, self-leadership, fit between individuals, tasks and technology framework

## Abstract

As AI becomes increasingly integrated into the workplace, understanding how prevailing multitasking practices interact with AI support to foster employee self-leadership is essential for enhancing organizational effectiveness. This study elucidates how the fit between multitasking and polychronicity among employees in organizations can synergistically influence their self-leadership within the context of AI empowerment. This study conducts two time-lagged survey studies using polynomial regression analysis, block variable analysis, and response surface methodology based on the “Fit Between Individuals, Tasks and Technology” (FITT) framework and the JD-R theoretical model. Study 1 examined the polychronicity-multitasking fit based on data collected from 116 employees at two time points in an AI company in China. Study 2 tested the mediating and moderating effect based on data of 188 employees from two other AI companies in China at three time points. The results show that congruence between polychronicity and multitasking predicts greater employee self-leadership compared to incongruence, and the higher the degree of congruence, the stronger the self-leadership. For incongruence, the “high-low” state promotes self-leadership better than the “low-high” state. We also reveal the mediating role of thriving at work and the moderating role of AI-empowered task processing between polychronicity-multitasking fit and self-leadership. For well-matched employees, AI serves as a facilitator of task processing, thereby enhancing employee self-leadership; whereas for mismatched ones, AI acts as an additional task burden or as a catalyst that exacerbates the existing imbalance, which impedes the motivation for self-leadership. These findings advance the understanding of self-leadership in multitasking contexts and provide valuable insights for organizations implementing AI tools. This study underscores the critical importance of aligning employees' work preferences with task demands to fully leverage the potential of AI empowerment.

## 1 Introduction

In modern organizations, the popularization and application of informatization, digitalization, and the vigorous development of Artificial Intelligence (AI) and Machine Learning (ML) have exerted disruptive changes to organization and employees (Chowdhury et al., 2023; Borges et al., 2021; Cheng et al., 2023; Makarius et al., 2020; Tang et al., 2023). In the fast-paced work environments, employees are often expected to juggle multiple tasks simultaneously, a demand that is further amplified by the integration of AI tools. For instance, an employee in a tech company may be tasked with managing several projects while utilizing AI-based tools to streamline their workload. This dynamic creates both opportunities and challenges, particularly in how employees manage their self-leadership in the face of increasing multitasking demands. Indeed, the status quo of a flatter, more open, and more autonomous human-machine coexistence increasingly highlights the pivotal role of employee self-leadership (Bakker et al., 2023), serving as a crucial factor in individual growth and development and sustainable human-machine team collaboration in the workplace (Stewart et al., 2019; Harari et al., 2021; Mueller and Niessen, 2019; Prikshat et al., 2023). Employees' self-leadership encompasses a comprehensive process of self-influence, wherein individuals spontaneously employ specific behavioral and cognitive strategies to proactively bridge gaps and attain desired objectives (Stewart et al., 2019). For example, employees might adopt self-leadership strategies like behavioral awareness to recognize task management issues, task motivation to stay focused, and constructive cognition to reframe challenges, continuously bridging the gap between their status and the desired goals. Regarding the antecedents of employee self-leadership, extant studies have identified a range of factors, predominantly focusing on single-dimensional variables related either to leadership or individual characteristics, such as personality characteristics (Furtner and Rauthmann, 2010; Houghton et al., 2004), emotional intelligence (Houghton J. D. et al., 2012), leadership training (Stewart et al., 1996), and various leadership styles (Andressen et al., 2012; Amundsen and Martinsen, 2015). Besides, several studies have examined the constructive role of person-job fit or person-environment fit in fostering employees career management behavior (Sirén et al., 2021; Abdalla et al., 2019), job engagement (Cai et al., 2018; Bui et al., 2017), proactive career behavior (Sylva et al., 2019). However, beyond the typical dyad elements fit, there is little known about how could dynamic interaction of multiple dimensions in the workplace (e.g., individual characteristic, work state and work-related frontier technology) shape employee self-leadership and its potential developing mechanism. To address this gap, this study takes the multidimensional fit perspective (Ammenwerth et al., 2006) and focus on the specific fit states in the context of human-machine interaction demonstrated by the prevalent multitasking state faced by employees and individuals' preferences for managing multiple tasks as well as task-aided AI technology.

The advent of AI technology in organizations, generally seen as an efficiency-enhanced tool, making employees more likely to be exposed to multitasking pressures (Prikshat et al., 2023), while also potentially changing their attitudes toward multitasking management. Specifically, its reshaping of the work content itself first necessitates employees to accomplish more tasks with less human capital in a highly competitive work state (Prikshat et al., 2023), which leads to a context characterized by the simultaneous engagement in multiple tasks and frequent task-switching, demanding employees' constant attention shifts (Kapadia and Melwani, 2021), i.e., multitasking. Besides, featured as an element of employee autonomy, polychronicity is defined as an individual's preference for repetitive task-switching without interruption and viewed as an individual trait resource to cope with the high demands in the workplace (Kirchberg et al., 2015). Present studies have found mixed effects of multitasking and polychronicity on employees' emotions, attitudes, behaviors, and performance (Kirchberg et al., 2015; Peifer and Zipp, 2019; Kapadia and Melwani, 2021; Sanderson et al., 2013; Howard and Cogswell, 2022). Some studies have further explained such mixed results by identifying the “supply and demand relationship” of multitasking. For example, polychronicity-multitasking fit significantly influences employees' job satisfaction (Hecht and Allen, 2005), creativity (Madjar and Oldham, 2006), organizational self-esteem (Hui et al., 2010). In this study, we will first adopt a person-job fit perspective to explore whether polychronicity-multitasking fit could shape employee self-leadership and how.

**Figure 1:**
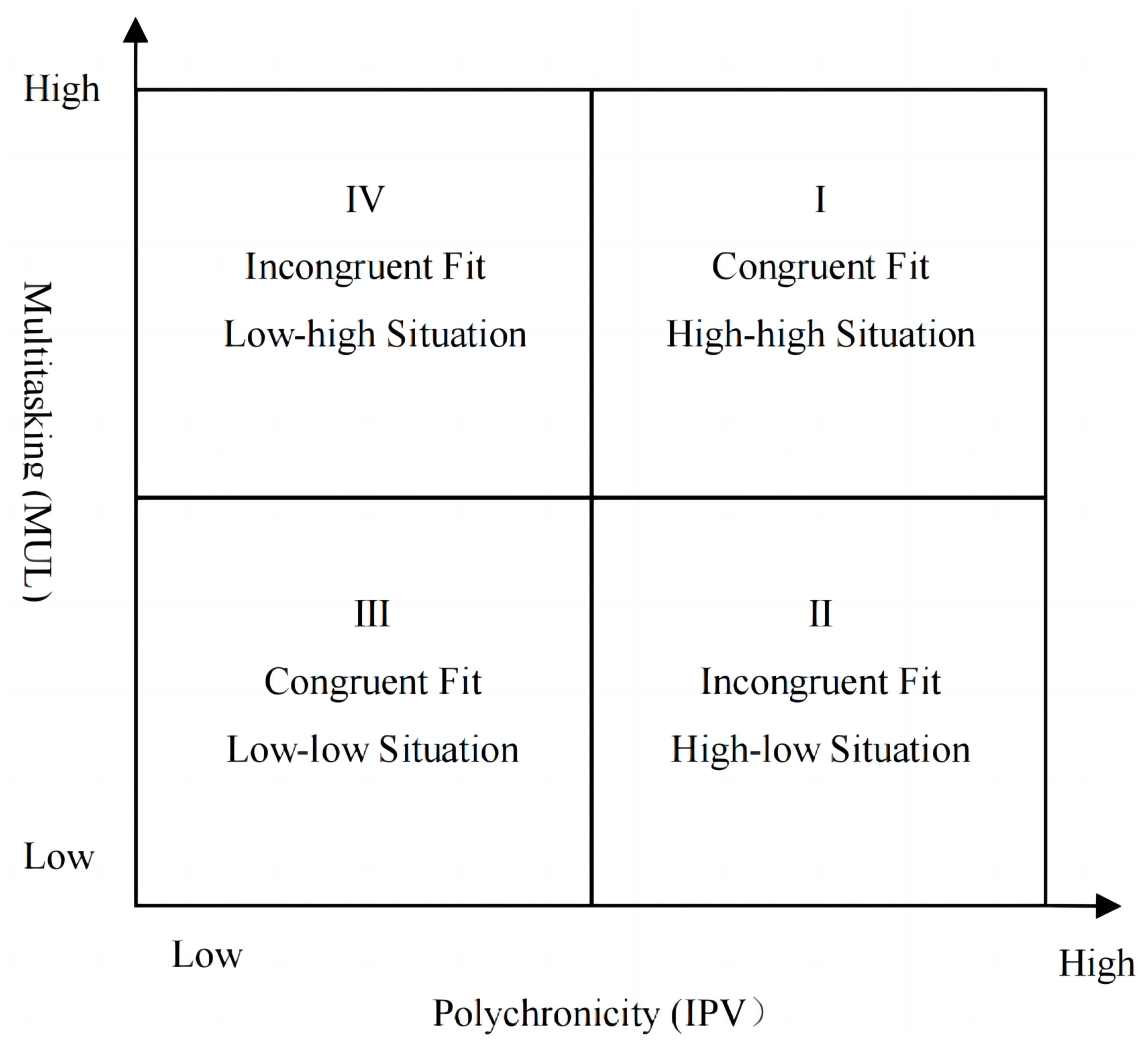
Polychronicity-multitasking fit states.

We extend the multidimensional fit literature further by examining the conditional effect of AI-empowered task processing on the focal relationship between polychronicity-multitasking fit and employee self-leadership. Despite being exposed to the similar AI tools in the workplace, different employees exhibit varying degrees of perceived empowerment in task processing (Cheng et al., 2023). Previous research has shown that the integration of artificial intelligence into work tasks can have diverse impacts on employees' attitudes and behaviors, including job satisfaction, career adaptation, and service innovation behaviors, among others (Presbitero and Teng-Calleja, 2022; Liang et al., 2022). However, most research has predominantly focused on individual characteristics, subjective perceptions and interactions with AI (Tang et al., 2023), neglecting to explore the diverse roles that AI plays for employees with varying job characteristics and work states. Although a remarkable study conducted by Verma and Singh (2022) has demonstrated the impact of AI-enabled job characteristics on employees' innovative work behavior, it has not explicitly investigated the influence of specific dynamic work states on employees' self-leadership. To draw more in-depth insights, we focus on four prototypical working states of polychronicity-multitasking fit from a more granular perspective, namely “high-high” and “low-low” of congruent fit, as well as “high-low” and “low-high” of incongruent fit. Figure 1 illustrates the four prototypical combinations of congruent and incongruent polychronicity-multitasking fit. We argue that for employees in congruent fit between polychronicity and multitasking, perceived AI empowerment serves as a facilitator of task processing, thereby enhancing employee self-leadership. However, for incongruent ones, AI in the task processing acts as an additional task burden or as a catalyst that exacerbates the existing imbalance, which will impede the motivation for employee self-leadership.

To provide insightful elucidation on whether and how the polychronicity-multitasking fit of individuals could synergistically shape employees' self-leadership under various levels of AI-empowered task processing, this study based on the “Fit between Individuals, Tasks and Technology” (FITT) framework (Ammenwerth et al., 2006) to investigate the specific impacts of the combinations of three elements (i.e., polychronicity, multitasking and AI-empowered task processing) on employee self-leadership and the underlying mechanism. The FITT framework highlights the alignment between three key elements in the workplace: individual characteristics (i.e., skills, preferences), task demands (i.e., workload, complexity), and technological tools (i.e., AI, automation systems). The framework posits that optimal outcomes are achieved when there is a strong fit among these elements. By further introducing the Job Demands-Resources (JD-R) theoretical model (Bakker et al., 2005) into the research framework, we emphasize the dual paths of demands and resources of three elements in the FITT framework. Drawing on assumptions of “buffering” and “coping” in JD-R model, this study investigates and discusses the interactive dynamics between various combinations of polychronicity, multitasking and AI-empowered task processing as job demands and resources, thereby unfolding their specific impact on employees' self-leadership. Moreover, it is noteworthy that the implementation of AI, while granting employees an enhanced level of autonomy in their tasks, may also engender detrimental consequences such as diminished perceived control and self-efficacy (Hu and Min, 2023; Kellogg et al., 2020; Petriglieri et al., 2019; Tang et al., 2023). Its impact on self-leadership for employee with different work states exhibits significant heterogeneity. Therefore, this study clarifies the specific attributes (i.e., as demands or resources) of AI in different contexts (i.e., four prototypical states of polychronicity-multitasking fit) and delineates the boundary conditions under which polychronicity-multitasking fit can exert its most positive influence on employee self-leadership.

Our theorizing makes three broad contributions. First, this paper contributes to the existing literature on JD-R theory (Bakker and Demerouti, 2017) by illuminating the distinct impact on self-leadership resulting from diverse matching states of three multitasking-related elements (i.e., fit between polychronicity, multitasking, and AI-empowered task processing) in the era of artificial intelligence. We present the four prototypical states of polychronicity-multitasking fit based on JD-R theory, which vividly illustrate the dual pathways of work resources (i.e., polychronicity) and demands (i.e., multitasking), as well as the “buffering” and “coping” effects among them on employees' self-leadership. This strongly resonates with Howard and Cogswell (2022) calling for “investigate the effects of excessive and deficient multitasking pressures, which may differentially produce motivation.” Moreover, our analysis identified a deeper JD-R theoretical tension in AI empowerment at workplace (Bakker et al., 2023). We reveal that whether AI empowerment is a “resource” as a “co-pilot” in task processing, or a “demand” as an additional burden or imbalance booster for employees needs to be discussed specifically according to different polychronicity-multitasking fit states. This has further enriched the theoretical insights of JD-R, especially from the perspective of the specific properties of burgeoning AI technology at work.

Second, by innovatively introducing the FITT framework (Ammenwerth et al., 2006) into the field of organizational management, this study expands the cutting-edge technological dimension in the work environment beyond the traditional dyad “person-job fit” framework (Chowdhury et al., 2023; Makarius et al., 2020), which also resonates with Howard and Cogswell (2022) calling for directly integrating P-E fit theory into the polychronicity research. This study comprehensively explores the developing veins and discrepant impacts of different polychronicity-multitasking fit on employee self-leadership within varying degrees of AI empowerment, with a particular emphasis on the transformative impacts of cutting-edge technologies on individual self-growth (Vrontis et al., 2022). Although previous research on polychronicity-multitasking fit has laid a helpful foundation for our exploration, the focus on the prevailing frontier technology environment in the organization such as AI assisting multitasking is coarse-grained and limited. We advance the literature by illustrating the ambivalent impact of AI-empowered task processing on employees with various work states, with the objective of determining the optimal condition for leveraging AI assistance to foster employee self-leadership development. In this regard, this study calls attention to zooming in the intricate and contingent value and challenges of artificial intelligence for employees with different work states in the workplace.

Third, this study extends the self-leadership literature by establishing the linkage between polychronicity-multitasking fit and employee self-leadership, and by identifying the role of AI and its influencing mechanism. Previous studies have been limited to exploring how individual characteristics and leadership traits influence employees' self-leadership, largely neglecting the importance of the interactive perspective of employees' working state and cutting-edge working environment (Bakker et al., 2023). In this paper, we propose various combinations of polychronicity, multitasking, and AI-empowered task processing, based on the FITT framework, would trigger diverse levels of self-leadership among employees. Moreover, by examining the mediating role of thriving at work in the relationship between polychronicity-multitasking fit and self-leadership, this study enhances the research on the socially embedded model of thriving at work and further expands the exploration of the motivational process of self-leadership development (Goh et al., 2022; Harari et al., 2021). This paper offers a novel and multi-dimensional perspective on fostering individual self-leadership within organizations empowered by emerging artificial intelligence and is the first step to a new research stream that explore the dynamic perspective of self-leadership development.

## 2 Theoretical background and hypotheses development

### 2.1 Theoretical background and research framework

Person-job fit refers to the alignment between an individual's abilities, values, and the demands or characteristics of the job (Kristof-Brown et al., 2005). With the ongoing transformation of organizational environments driven by digital and intellectual technologies, the conventional “person-job fit” model inevitably fails to account for the disruptive effects on work environments and employees' perceptions that may arise from technological interactions (Kristof-Brown et al., 2005; Vrontis et al., 2022). From a forward-looking and comprehensive perspective, this study adopts the “Fit between Individuals, Tasks and Technology” (FITT) framework proposed by Ammenwerth et al. (2006), which is innovatively integrated into the realm of organizational management within the context of artificial intelligence change. This breakthrough surpasses the limitations of previous two-dimensional matching models and unveils a holistic panorama of employee development through dynamic interactions among three key elements (Makarius et al., 2020). We aim to systematically integrate the multiple elements of employees and dynamically changing work environments, to construct a comprehensive theoretical framework that is compatible with other related organizational management theorizing, bridges theory and practice, and keeps pace with technological advancements.

The Job-Demand-Resource (JD-R) theory (Bakker et al., 2005) was introduced into the research framework to explain the effectiveness of each element and their interactive effects, thereby facilitating a meticulous delineation of employee self-leadership development in the era of artificial intelligence. In this FITT framework, the “task” dimension specifically addresses the inherent complexities of multitasking within technology-driven work environments (Howard and Cogswell, 2022). It is regarded in the JD-R model as an indication of burnout manifested through excessive work demands (Kapadia and Melwani, 2021). The “individual” dimension is represented in the framework as a personal trait factor corresponding to multitasking, i.e., polychronicity (Sanderson et al., 2013). According to the JD-R model, employees' autonomous response to work demands is considered a proactive factor that elicits a gain effect as an individual psychological resource at work (Kapadia and Melwani, 2021). Simultaneously, drawing on the “buffering” and “coping” assumptions of the JD-R model, the dynamic interaction between polychronicity and multitasking would also generate diverse effects (Howard and Cogswell, 2022). The “technology” dimension focuses on the degree of AI-empowered task processing in the workplace (e.g., AI assists task planning or content generation, or AI assists task information data collation and analysis), and its attribute in the JD-R model is more intricate (Vrontis et al., 2022). Its disruptive impact on the work environment and employee growth exhibits as a “double-edged sword” within varying states of polychronicity-multitasking fit.

As illustrated in Figure 1, this study aims to investigate: (1) whether there is a higher level of self-leadership among employees when there is congruence between polychronicity and multitasking, as opposed to incongruence; (2) whether the “high-low” state is more effective than the “low-high” state in fostering employees' self-leadership within two types of incongruence; (3) what is the underlying mechanism through which the polychronicity-multitasking fit could exert its influence on self-leadership; (4) under different levels of AI empowerment, what are the significant changes in the effects of congruent and incongruent matching on employee self-leadership. In summary, within the FITT framework and grounded on the JD-R theoretical model, this study will comprehensively explore the impacts and mechanisms of different polychronicity-multitasking fit on employees' self-leadership development, and further examine how AI empowerment affects these results.

### 2.2 Polychronicity-multitasking fit and employee self-leadership

The JD-R model's “dual-path” and “buffering” hypotheses propose that employees who are congruently matched can effectively manage their multitasking requirements through autonomous multitasking preferences (Mattarelli et al., 2015), thereby enabling them to cope with demanding tasks while mitigating the burnout caused by high levels of multitasking pressure (Anser et al., 2022). In this process, the effective utilization of employees' intrinsic motivation to proactively initiate a series of behavioral actions can bridge the gap between the reality and desired objectives, thereby significantly fostering the development of employee self-leadership (Stewart et al., 2019). However, in the mismatched condition, employees are confronted with a substantial incongruity between their multitasking preferences and the prevailing work requirements, thus constraining individuals' ability to stimulate their intrinsic motivation and reach predetermined objectives (Howard and Cogswell, 2022). Consequently, this negatively predicts the development of employee self-leadership which focused on “autonomy” and “goal-setting” (Stewart et al., 2019). The present study posits employees' self-leadership is higher when there is congruence rather than incongruence in polychronicity-multitasking fit.

However, it is worth noting that the impact of varying levels of congruent matching exhibits significant disparities. According to “coping” hypothesis of JD-R model, employees operating in highly challenging work environments demonstrate a higher capacity to activate and mobilize their work resources to effectively accomplish task goals (Bakker et al., 2007). As a result, employees who are congruently matched in a “high-high” state could proactively tap into their motivational potential to access internal or external work resources when confronted with demanding multitasking requirements (Lesener et al., 2019). They are more inclined to spontaneously meet their high work standards and strict task requirements, fully engage in the working process, and actively explore the available resources for their tasks (Zhang and Parker, 2019). In essence, their preference for autonomy in multitasking prevails as a means to cope with high work pressure, thereby predicting a heightened level of self-leadership (Cranmer et al., 2019). However, in the “low-low” congruent state, employees are provided with less robust incentives from external work challenges and thus exhibit diminished motivation toward work autonomy (Zhang and Parker, 2019). Consequently, the ability of employees to access and utilize work resources for “reachable” task management objectives will be limited, resulting in a reduced impact on self-leadership compared to that in the “high-high” state. Thus, the following hypothesis is proposed:

**Hypothesis 1a:** The employee self-leadership is higher when there is congruence rather than incongruence in polychronicity-multitasking fit, and the greater the degree of congruence, the higher the level of self-leadership.

The impact of the incongruent polychronicity-multitasking fit varies in terms of its specific effects on self-leadership, depending on whether it is a “high-low” or “low-high” state. This study proposes that the “low-high” state has a less favorable effect on employees' self-leadership development compared to the “high-low” state. Present studies indicated that intrinsic autonomy is of greater importance than extrinsic goal motivation in the development of self-leadership (Bakker et al., 2023; Stewart et al., 2019). The positive impact of employees' high polychronicity as an autonomous work resource on self-leadership is more pronounced in the “high-low” state compared to the “low-high” state. Moreover, despite the presence of an “oversupply” of individual trait resources in the “high-low” state, employees' multitasking demands can be effectively accommodated by their existing multitasking preferences, resulting in a relatively attenuated burnout effect (Zhang and Parker, 2019). In contrast, the “low-high” state, characterized by intense work pressure and limited individual resources, may exert a more pronounced impeding effect on employee self-leadership (Harari et al., 2021). Moreover, in the “low-high” state, excessive multitasking pressure will further aggravate the depletion of employees' work resources (Schaufeli, 2017). According to the JD-R theory, when employees experience a loss of resources, they would like to take defensive measures to prevent further resource depletion (Bakker and Demerouti, 2017). Consequently, employees would exhibit a greater inclination toward risk aversion, reduced commitment, diminished work motivation and self-leadership motivation in such circumstances. Therefore, the following hypothesis is proposed:

**Hypothesis 1b:** When there is incongruence in polychronicity-multitasking fit, employee self-leadership is higher in the “high-low” state than in the “low-high” state.

### 2.3 The mediating role of thriving at work

Thriving at work encompasses the positive work states of “vitality” and “learning” that employees experience in their workplace (Spreitzer et al., 2012). First, for the affective dimension, the “vitality” experience signifies the overall energy exerted by employees at work, which fosters individual motivation to actively engage in reflective feedback and self-improvement (Goh et al., 2022), thereby encouraging them to actualize their utmost potential for self-fulfillment and proactively bridge the gap between reality and expectations (Spreitzer et al., 2012). The “vitality” experience, as the positive work resource in the JD-R model, thus motivates employees to actively pursue their goals and growth (Walumbwa et al., 2018), leading to more proactive self-leadership behaviors. Second, for the cognitive dimension, the employees' “learning” experience entails the acquisition of knowledge and skills, as well as the enhancement of individual capabilities (Goh et al., 2022). The acquisition of richer work skills and experiences as implicit work resources will enable employees to better adapt to the dynamically changing work environment, access more external resources, and employ more effective self-regulation strategies to achieve higher working goals (Shahid et al., 2021; Alikaj et al., 2020). In addition, the “learning” process engenders a virtuous cycle that perpetually enhances employees' competence and expertise, accommodating their intrinsic needs for autonomy and competence (Ryan and Deci, 2017), and its positive shaping of self-leadership will be more prominent with iterative and updated process of learning and experiencing (Prem et al., 2017). Thus, employee' perception of thriving at work will positively influence their self-leadership development.

How can employees' perception of thriving at work be enhanced in a multitasking context? According to the Socially Embedded Model of Thriving (SEMT; Spreitzer et al., 2005), the antecedents of employees' thriving at work can be classified into two categories, i.e., work demands and work resources. Based on JD-R's “dual-path” and “buffering” hypotheses, congruently matched employees, though facing high levels of multitasking pressure, can effectively utilize their autonomous work resources, i.e., polychronicity, to soundly meet the high requirements (Kirchberg et al., 2015). This enables them to efficiently handle the intense demands of multitasking and fully leverage their intrinsic motivation, thereby fostering a state of high productivity and contributing to a sense of thriving at work (Spreitzer et al., 2012). In addition, the “coping” assumption suggests that employees in a “high-high” state, compared to a relatively “relaxed” environment in “low-low” state, are stimulated to actively seek internal and external work resources in response to the exterior stimuli of demanding multitasking requirements (Bakker et al., 2007). Thus, employees are more inclined to spontaneously meet the high standards in the workplace, fully engage in their work, positively leverage their resources, and take the initiative to create learning opportunities and clear directions for individual growth, which predicts higher levels of thriving at work (Shahid et al., 2021; Xanthopoulou et al., 2009). Apart from that, in both states of incongruent matching, “oversupplied” multitasking in “high-low” state offers a more abundant pool of potentially activatable work resources compared to the “low-high” state (Lesener et al., 2019). As a result, employees tend to demonstrate elevated work pursuit and self-perception, leading to a relatively positive state characterized by active learning and enhanced vitality (Nawaz et al., 2020). This, in turn, would predict higher levels of employee self-leadership. In summary, compared to incongruence, congruent matching exerts a more pronounced positive impact on employee self-leadership through thriving at work. Among them, “high-high” state predicts a stronger positive effect on self-leadership via thriving at work. When it comes to incongruence, the positive effect of “high-low” state on self-leadership via thriving at work is more potent than that of “low-high” state. Thus, the following hypothesis is proposed:

**Hypothesis 2:** Thriving at work mediates the relationship between polychronicity-multitasking fit and employee self-leadership.

### 2.4 The moderating role of AI-empowered task processing

As AI is increasingly embedded in the traditional organizational context, whether the complex human-computer interaction system is an enabling or a disabling factor for organizations and employees amidst the high pressure of multitasking necessitate in-depth investigation considering employees' traits, work states, and their interaction effects (Tang et al., 2023). Employees of congruent fit between polychronicity and multitasking are more able to work smoothly and thus meet higher autonomy and competence needs (Ryan and Deci, 2017). Moreover, the assistance of AI technology has led to a more substantial enhancement in work autonomy, enabling individuals to access a wider range of positive resources both internally and externally to meet higher work demands and engage in self-development (Prikshat et al., 2023), which in turn potently inspire self-leadership. For employees of incongruent fit, however, under the influence of advanced intelligent technologies, the impeding effect on employees' individual growth and self-leadership resulting from such incongruous states would be further accentuated (Aleem et al., 2023). Specifically, from the negative perspective of work demand, organizational technological transformation may lead to an augmentation in employees' workload and psychological stress (Cheng et al., 2023; Tang et al., 2023). In particular, the acquisition and application of emerging technologies impose additional cognitive and practical demands on employees who already struggle with multitasking, necessitating their heightened inputs to meet the new threshold of job skills (Prikshat et al., 2023). This might significantly reshape the original work situation, increase employees' job burden, and even threaten their employment stability and career progression (Chowdhury et al., 2023). Thus, based on the JD-R model, cutting-edge technology in the workplace is more of a vital work demand than a supplementary resource for employees of incongruent polychronicity-multitasking fit, which may hinder employees' self-leadership development. In summary, the following hypothesis is proposed:

**Hypothesis 3a:** AI-empowered task processing positively moderates the relationship between the congruence in polychronicity-multitasking fit and employee self-leadership, and negatively moderating the relationship between incongruence in polychronicity-multitasking fit and employee self-leadership.

Despite both impeding self-leadership, employees in both types of incongruences tend to exhibit distinct adverse responses to the high levels of artificial intelligence. In the “high-low” state, embedded AI technology will further accentuate the imbalance status of work (Cheng et al., 2023), such as highlighting the low-challenging status quo of “resources outweighing demands” and amplifying employees' sense of powerlessness in their inability to effectively utilize resources to fulfill their needs (Tang et al., 2023). This engenders a heightened perception of ego depletion, thereby significantly impeding the development of self-leadership. However, under the “low-high” state, a high level of AI empowerment can provide employees with a certain degree of job autonomy and efficacy, even in the absence of individual initiative (Prikshat et al., 2023). Specifically, AI in task processing can assist them to fulfill multitasking demands efficiently (Chowdhury et al., 2023), which partially compensates for the lack of preference for polychronicity. In other words, although acknowledging that the positive effect of AI empowerment for employees in “low-high” incongruent state is limited, it still partially mitigates the hindering effect stemmed from such imbalanced status quo. Taken above together, the following hypothesis is proposed:

**Hypothesis 3b:** When there is incongruence in polychronicity-multitasking fit, with the moderating effect of AI empowered task processing, employee self-leadership is higher in the “low-high” state than in the “high-low” state.

**Figure 2:**
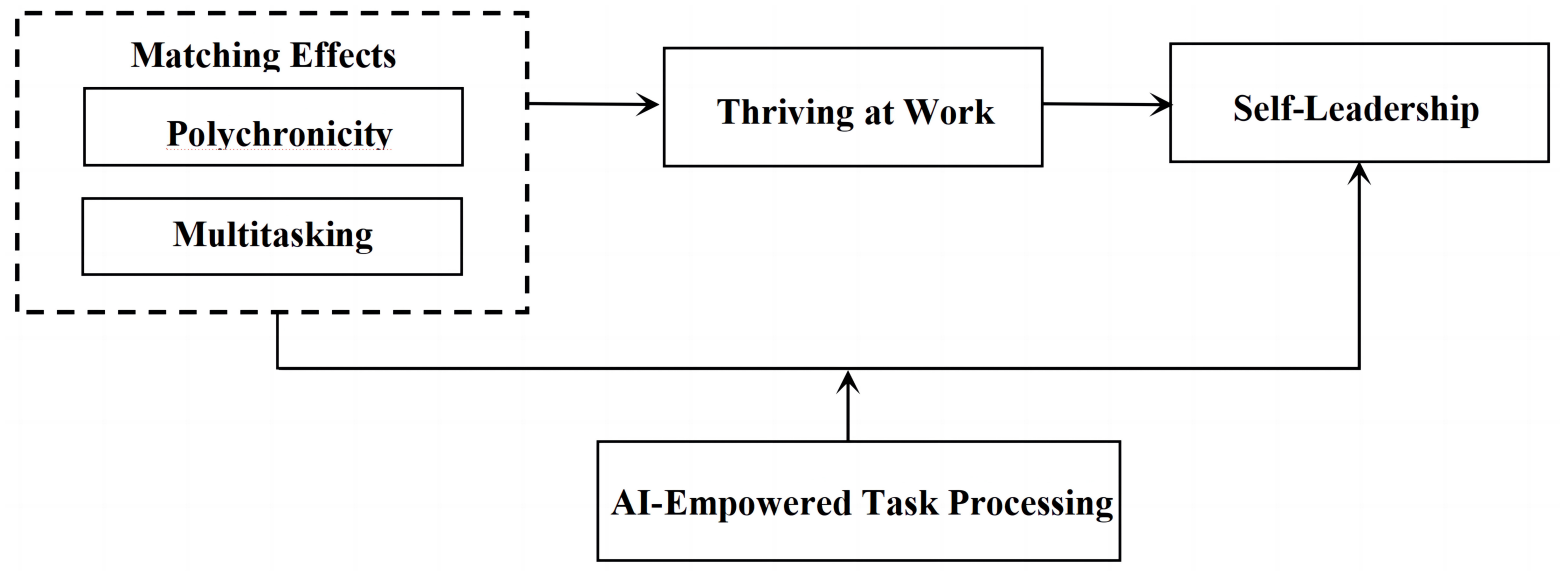
Theoretical model.

Figure 2 demonstrates the theoretical model of the study based on above research hypotheses.

## 3 Study1

### 3.1 Method

#### 3.1.1 Participants and procedures

Our survey was conducted in the headquarter of a large AI technology company in Chengdu, China. We obtained the roster of employees who were willing to participate in the survey from the human resource management department, together with their email addresses. We sent emails to those employees, introduced the survey purpose and procedure, and guaranteed the voluntariness and anonymity of the survey. We iterated the results obtained from the study would be intended solely for academic research purposes, with strict adherence to confidentiality protocols that prohibit any disclosure of information to third parties. To eliminate common method bias (Podsakoff et al., 2003), we adopted a multi-wave (i.e., Time 1 and Time 2) survey design with a 2-week interval. For two time points' data matching, we used the last four digits of the employees' cell phone tail numbers. The specific procedures were as follows: (1) At Time 1 (T1): 140 questionnaires were distributed and 124 were returned, in which employees provided their demographic information (i.e., age, gender, education, tenure) and evaluated their perceived levels of polychronicity and multitasking; (2) At Time 2 (T2): 124 questionnaires were distributed and 116 were returned, in which employees evaluated their own self-leadership in the workplace. To incentivize participation and improve the response quality, participants would be rewarded with a monetary reward upon completion of the Time 1 survey, along with an additional monetary reward upon completing the Time 2 survey. Through screening and matching, 116 valid questionnaires were finally obtained, with a valid answer rate of 82.86%. Among them, 69.0% were male, bachelor degrees and above accounted for 89.6%, people aged 26–40 were 79.3%, and 57.8% had more than 3 years of job tenure.

#### 3.1.2 Measures

The variables in Study 1 were all measured with well-established scales, which has been proven to have good reliability and validity. English scales were translated into Chinese using the “translation/back translation” procedure (Brislin, 1980). Participants expressed the degree of their agreement with the given statements using a 7-point Likert scale from 1 (strongly disagree) to 7 (strongly agree).

##### 3.1.2.1 Polychronicity

Employees' polychronicity was measured using the Inventory of Polychronic Values (IPV) scale developed by Bluedorn et al. (1999), which consists of 10 items. A sample item included “We like to juggle several activities at the same time.” Cronbach's alpha was 0.845.

##### 3.1.2.2 Multitasking

This variable was measured using a ten-item measurement adapted from the multitasking scale of Bluedorn et al. (1999). Following Hecht and Allen's (2005) method of modifying the items to align with work characteristics rather than individual preferences, we employed a multitasking scale in line with the polychronicity preference scale. A sample item included “This job demands that I juggle several activities at the same time.” Cronbach's alpha for this scale was 0.890.

##### 3.1.2.3 Self-leadership

We utilized the short version of the self-leadership scale developed by Houghton J. et al. (2012) to measure employees' self-leadership. This scale consists of three dimensions, namely, behavioral awareness and decision making, task motivation, and constructive cognition, and each dimension included three items. A sample item included “I establish specific goals for my own performance.” Cronbach's alpha was 0.867.

##### 3.1.2.4 Control variables

Extant research has identified that gender, age, tenure, education, and other demographic information would exert an influence on employee self-regulated attitudes and behaviors during organizational technological change (e.g., Tang et al., 2023; Chowdhury et al., 2023). Thus, we included employee gender, age, tenure, education level as control variables in study 1.

#### 3.1.3 Analytical strategy

Study 1 employed polynomial regression and response surface methodology (Edwards and Parry, 1993; Jansen and Kristof-Brown, 2006) to test hypothesis 1a and hypothesis 1b. Prior to the polynomial regression and response surface analysis, it should be first examined that the proportion of “incongruent” samples was determined to be more than 10%, as recommended by Shanock et al. (2010). The polynomial regression model developed in Study 1 is presented below:


(1) $$\begin{array}{l} SL=b_{0}+b_{1}IPV+b_{2}MT+b_{3}IPV^{2}+b_{4}IPV\times MT\\ \;+b_{5}MT^{2}+e \end{array}$$

*SL* stands for employees' self-leadership, *IPV* stands for polychronicity, *MT* stands for multitasking, *IPV*^2^ denotes the squared term of polychronicity, *IPV*×*MT* implies the cross-product term of polychronicity and multitasking, and *MT*^2^ is the squared term of multitasking. *b*_1_*b*_5_ are the regression coefficients; *e* denotes the residuals of the regression equation. Polychronicity, multitasking, and self-leadership were all mean-centered (Hofmann et al., 2000) in study 1.

SPSS 26.0 was used to calculate the polynomial regression coefficients and the response surface' slopes and curvatures along the congruent and incongruent lines, after which we draw the three-dimensional response surface plots accordingly. If the *F* value of the polynomial regression Equation 1 is significant, the model fits well. If Δ*R*^2^ is significant after adding three higher-order terms (*IPV*^2^*IPV*×*MTMT*^2^), and the coefficients of at least one of the higher-order term are significantly different from 0, then it indicates the model is suitable for the polynomial regression analysis. This allows for the subsequent construction of characteristic data and the delineation of the three-dimensional response surface plot.

According to Edwards and Cable (2009), the significant characteristic data of the three-dimensional surfaces needs to be estimated. To test the effect of congruent matching in Study 1, if the curvature of the response surface (*b*_3_−*b*_4_+*b*_5_) along the incongruence line (*IPV* = −*MT*) is significantly negative and the 95% confidence intervals for the slope of the first principal axis, *p*_11_, includes 1, then it suggests that the level of employee self-leadership is stronger in the case of congruence compared to the case of incongruence and thus hypothesis 1a could be partially supported. If the curvature *b*_1_+*b*_2_ of the response surface along the congruence line (*IPV* = *MT*) is significantly >0 and the curvature *b*_3_+*b*_4_+*b*_5_ along the congruence line (*IPV* = *MT*) is not significant, then it implies that employee self-leadership is higher in the case of a “high-high” congruent state as compared to the case of a “low-low” state and thus a congruence effect in hypothesis 1a would be supported. When testing the incongruence effect of Study 1, if the curvature of the response surface (*b*_1_−*b*_2_) along the incongruence line (*IPV*= −*MT*) is significantly >0 and the side shift (*b*_2_−*b*_1_)/[2 × (*b*_3_−*b*_4_+*b*_5_)] along the incongruence line (*IPV* = −*MT*) proposed by Edwards and Parry (1993) is also significantly >0, it indicates that employees' self-leadership is higher in the “high-low” state compared to the “low-high” state and thus hypothesis 1b could be supported.

### 3.2 Results

#### 3.2.1 Confirmatory factor analyses

Study 1 conducted confirmatory factor analyses (CFAs) of the three key variables using MPLUS 8.7, and the results are shown in Table 1. Due to the large number of scale items for polychronicity and multitasking and the relatively small sample size in Study 1, the sample data were packaged according to the recommended practice of Mathieu and Farr (1991). As shown in Table 1, the three-factor model fit was significantly better than the other alternative models (χ^2^/*df* = 1.368, *RMSEA* = 0.056, *SRMR* = 0.059, *CFI* = 0.924, *TLI* = 0.915). It indicates that three key variables in the model of the present study exhibited good discriminant validity and that there was not a severe common method bias, which necessitated the establishment of a three-factor model.

**Table 1:** Results of confirmatory factor analysis of Study 1.

**Models**	**χ^2^**	***df***	**χ^2^*/df***	***RMSEA***	***SRMR***	***CFI***	***TLI***
Three-factor model (IPV, MT, SL)	310.570	227	1.368	0.056	0.059	0.924	0.915
Two-factor model (IPV+MT, SL)	383.580	229	1.675	0.076	0.076	0.859	0.845
One-factor model (IPV+MT+SL)	516.172	230	2.244	0.104	0.095	0.740	0.713

N = 116. IPV, polychronicity; MT, multitasking; SL, self-leadership. +: factor added together.

#### 3.2.2 Descriptive statistics

Table 2 presents the means, standard deviations, correlation coefficients, and Cronbach's alpha for the key variables in Study 1. As shown in Table 2, polychronicity was significantly positive associated with employee self-leadership (*r* = 0.678, *p* < 0.01). Multitasking was also significantly and positively related to employee self-leadership (*r* = 0.386, *p* < 0.01). This has provided initial support for our hypotheses. The Cronbach's alpha for all variables were above of 0.85, indicating that all scales have high internal consistency.

**Table 2:** Mean, standard deviation, and correlations of all variables of Study 1.

**Variable**	**Mean**	***SD***	**1**	**2**	**3**	**4**	**5**	**6**	**7**
1. Gender	1.310	0.465							
2. Age	2.210	0.763	0.038						
3. Education	2.920	0.399	0.037	0.110					
4. Tenure	2.860	1.257	0.193^*^	0.456^**^	−0.004				
5. Polychronicity	4.597	0.890	0.147	0.180	−0.094	0.098	(0.845)		
6. Multitasking	4.694	0.895	0.097	0.065	−0.072	0.141	0.855^**^	(0.890)	
7. Self-leadership	4.959	0.655	−0.046	0.238^*^	−0.083	0.012	0.678^**^	0.386^**^	(0.867)

N = 116. Gender: male = 1, female = 2. Age: 18–25 = 1, 26–30 = 2, 31–40 = 3, 41–50 = 4, above 50 = 5. Education: middle school = 1, senior high school = 2, bachelor degree = 3, master degree = 4, doctor degree and above = 5. Tenure: within 1 year = 1, 1–3 years = 2, 3–5 years = 3, 5–10 years = 4, 10 years and above = 5. Cronbach's alphas are shown in parentheses along the diagonal. SD, standard deviations. ^*^p < 0.05, ^**^p < 0.01.

#### 3.2.3 Hypotheses testing

In Study 1, using SPSS 26.0, results showed that the proportion of “incongruent” samples was as high as 30.17%, which exceeded the 10% criterion (Shanock et al., 2010). The polynomial regression results are shown in Table 3. The results show that after adding three higher-order terms (i.e., *IPV*^2^, *IPV*×*MT, MT*^2^), Δ*R*^2^in Model 3 increased significantly (Δ*R*^2^ = 0.083, *p* < 0.001). The coefficients of the three higher-order terms were all significantly different from 0, thereby substantiating the suitability of the model for polynomial regression analysis and response surface analysis.

**Table 3:** Results of polynomial regression analysis of Study 1.

	**Self-leadership**		
	**Model 1**	**Model 2**	**Model 3**
***Intercepts***	5.171^***^	3.274^***^	5.537^***^
**Control variable**			
Gender	−0.042	−0.237^***^	−0.176^*^
Age	0.264^***^	0.054	0.027
Education	−0.190	−0.021	0.006
Tenure	−0.064	−0.006	0.023
**Polynomial variable**			
Polychronicity (*IPV*)		0.958^***^	0.221^***^
Multitasking (*MT*)		−0.522^***^	−0.082
*IPV*^2^			−0.112^***^
*IPV*×*MT*			0.195^***^
*MT*^2^			−0.116^***^
*R*^2^	0.083	0.631	0.715
Δ*R*^2^	0.083	0.548^***^	0.084^***^
Δ*F*	2.498	80.929^***^	10.412^***^
***Congruence line (*** **IPV** ***=*** **MT** ***)***			
Slope (*b*_1_+*b*_2_)			0.138^***^
Curvature (*b*_3_+*b*_4_+*b*_5_)			−0.033^**^
***Incongruence line (*****IPV** ***=** **–*****MT*****)***			
Slope (*b*_1_−*b*_2_)			0.303^**^
Curvature (*b*_3_−*b*_4_+*b*_5_)			−0.423^***^
Side-shift (*b*_2_−*b*_1_)/[2 × (*b*_3_−*b*_4_+*b*_5_)]			0.358^*^

N = 116. ^*^p < 0.05, ^**^p < 0.01, ^***^p < 0.001.

**Figure 3:**
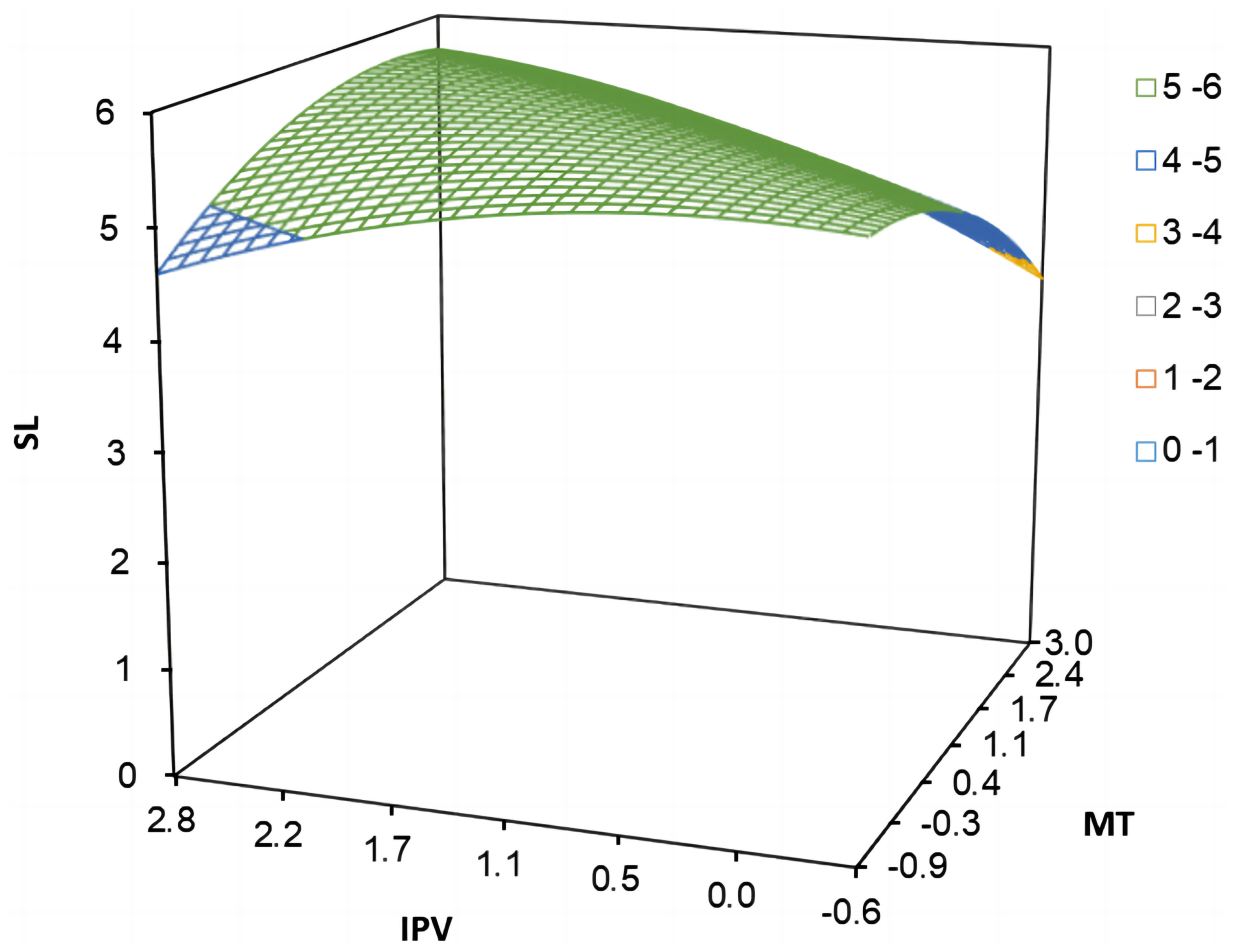
Response surface plot of polychronicity-multitasking fit and employee self-leadership.

Based on the polynomial regression and its matrix data, Study 1 plotted the three-dimensional response surface (see Figure 3) and estimated the significant characteristic data of the response surface (see Table 3). Specifically, the curvature of the response surface along the incongruence line (*IPV* = −*MT*) was significantly negative (*b*_3_−*b*_4_+*b*_5_ = −0.423, *p* < 0.001). The shape of the response surface along the incongruence line in Figure 3 suggests that employees would have higher levels of self-leadership when there was congruence in polychronicity-multitasking fit, providing initial support for hypothesis 1a. The bootstrapping bias-corrected analyses results of 10,000 resamples showed that the 95% *CI* (confidence intervals) for the first principal-axis slope, *p*_11_, was [0.785, 1.245], which included 1, thus there was no spindle deflection. Further, as shown in the visualized three-dimensional response surface plot of Figure 3, the value of employee self-leadership gradually increased along the longitudinal axis as the points along the incongruence line (*IPV* = −*MT*) gradually moved toward the congruence line (*IPV* = *MT*), which provided further support for hypothesis 1a. The slope of the response surface along the congruence line (*IPV* = *MT*) was significantly positive (*b*_1_+*b*_2_ = 0.138, *p* < 0.001), indicating that the positive effect of “high-high” congruent matching on employee self-leadership was more potent than that of “low-low” state. However, the curvature *b*_3_+*b*_4_+*b*_5_ along the congruence line (*IPV* = *MT*) was significantly negative (*b*_3_+*b*_4_+*b*_5_= −0.033, *p* < 0.01), it is necessary to further examine whether there are significant differences between the “high-high” and “low-low” states. Then we calculated the Z-Hat values for the “high-high” and “low-low” states, which were 5.554 and 4.765, respectively. The difference between the Z-Hat values for the “high-high” and “low-low” states was 0.789, with a 95% confidence interval that does not include 0 [95% *CI* = 0.510, 1.040]. This indicates that employee self-leadership is higher in the case of a “high-high” congruent state as compared to the case of a “low-low” state and thus a congruence effect in hypothesis 1a was supported. In view of the response surface plot in Figure 3, the points along the congruence line (*IPV* = *MT*) surface changed from small (i.e., “low-low”) to large (i.e., “high-high”), and the value of employee self-leadership on the longitudinal axis also increased accordingly. Therefore, hypothesis 1a was strongly supported.

The slope of the response surface along the incongruence line (*IPV* = −*MT*) was significantly positive (*b*_1_−*b*_2_ = 0.303, *p* < 0.01) for both types of incongruences in polychronicity-multitasking fit, suggesting that the positive impact of “high-low” state on employee self-leadership was more potent than that of “low-high” state. In addition, the side shift along the incongruence line (*IPV* = −*MT*) proposed by Edwards and Parry (1993) {i.e., (*b*_2_−*b*_1_)/[2 × (*b*_3_−*b*_4_+*b*_5_)]} was calculated to be 0.358, with a 95% *CI* of [0.094, 0.839], excluding zero. Thus, hypothesis 1b was supported. Further, given the response surface plot in Figure 3, the height of the surface along the incongruence line (*IPV* = −*MT*) was higher in the “IPV > MT” region than in the “MT > IPV” region, and the lowest value of employee self-leadership occurred in the “MT > IPV” region. It indicates that employee self-leadership was higher in the “high-low” state compared to the “low-high” state, which strongly supported hypothesis 1b.

## 4 Study 2

### 4.1 Method

#### 4.1.1 Participants and procedures

Samples for Study 2 were collected from two other AI technology companies headquartered in Chengdu, China. The procedures in study 2 prior to the survey were analogous to study 1. To minimize common method bias (Podsakoff et al., 2003), we adopted a multi-wave (i.e., Time 1, Time 2, and Time 3) survey design with a 2-week interval. For three time points' data, we matched them via the last four digits of the employees' cell phone tail numbers. The specific research procedures are as follows: (1) At Time 1 (T1): 224 questionnaires were distributed and 205 were returned, in which employees provided their demographic information (i.e., age, gender, education, tenure) and evaluated their polychronicity and perceived multitasking. (2) At Time 2 (T2): 205 questionnaires were distributed and 193 were returned, in which employees evaluated their perceptions of thriving at work and the perceived degree of AI-empowered task processing. (3) At Time 3 (T3): 193 questionnaires were distributed and 188 were returned, in which employees rated their self-leadership in the workplace. To activate participation and ensure response quality, participants would be rewarded with a monetary reward upon completion of the Time 1 survey, along with an additional monetary reward upon completing the Time 3 survey. Through a rigorous three-wave matching process, we excluded samples with missing data and obtained a total of 188 questionnaires, with a valid answer rate of 83.93%. Among them, 72.3% were male, 89.4% got bachelor degree or above, employees aged 26–40 accounted for 75.5%, and 55.3% had more than 3 years of job tenure.

#### 4.1.2 Measures

The variables in Study 2 were all measured with well-established scales, which has been proven to have good reliability and validity. English scales were translated into Chinese using the “translation/back translation” procedure (Brislin, 1980). Participants rated each item using a 7-point Likert scale from 1 (strongly disagree) to 7 (strongly agree). Three variables of multitasking, polychronicity and self-leadership were consistent with the measurement in Study 1, with Cronbach's alpha of 0.864, 0.893, and 0.862, respectively.

##### 4.1.2.1 AI-empowered task processing

This variable was measured using a five-item measurement adapted from the technology dimension of the task-technology fit scale developed by Goodhue and Thompson (1995). Considering that the original scale was designed for general science and technology in the workplace, this study based on the theme of AI technology supporting for task processing in the AI era adopted a shifted reference model to rephrase the items rationally. A sample item included “I can use AI technology to obtain relevant information in work tasks.” Cronbach's alpha for this scale was 0.748.

##### 4.1.2.2 Thriving at work

We employed a 10-item scale developed by Porath et al. (2012) to measure the perceived thriving at work, which consists of two dimensions, learning and vitality. An example item in the learning dimension featured “I continue to learn more as time goes by.” A sample item in the vitality dimension included “I feel alive and vital.” Cronbach's alpha was 0.856.

##### 4.1.2.3 Control variables

Consistent with Study 1, Study 2 included employees' gender, age, education, and tenure as control variables.

#### 4.1.3 Analytical strategy

For the mediation effect test of Study 2, following the approach proposed by Edwards and Cable (2009), we calculated a “Block Variable” by multiplying the original values of each polynomial regression variable with their respective regression coefficients and then summing them. We employed bootstrap-based statistics in MPLUS 8.7 to calculate the moderating effect estimates and constructed 95% bias-corrected confidence intervals (Hayes, 2009). Hypothesis 2 would be supported if the 95% *CI* for the indirect effect excludes 0.

For the test of the moderating effect, AI-empowered task processing (*AI*) was added as a moderator to construct a polynomial regression model with moderating effects in Equation 2:


(2) $$\begin{array}{c} SL=b_{0}+b_{1}IPV+b_{2}MT+b_{3}IPV^{2}+b_{4}IPV\times MT+b_{5}MT^{2}\\ \;+b_{6}AI+b_{7}AI\times IPV+b_{7}AI\times MT+b_{8}AI\times IPV^{2}\\ \;+b_{9}{AI\times IPV\times MT+b}_{10}AI\times MT^{2}+e \end{array}$$

IfΔ*R*^2^ of Equation 2 is significant after adding five higher-order terms of moderating variables (i.e., *AI*×*IPV*, *AI*×*MT*, *AI*×*IPV*^2^, *AI*×*IPV*×*MT*, *AI*×*MT*^2^), it indicates that moderating effect is supported. Moreover, the high and low groups of AI empowerment were separated based on the mean value plus or minus one standard deviation of the AI-empowered task processing. The curvature and slope of the response surface were examined, respectively, to compare the differences in polynomial regression coefficients between the high and low groups. We also visualized the three-dimensional response surface plot to further test hypothesis 3a and hypothesis 3b.

### 4.2 Results

#### 4.2.1 Confirmatory factor analyses

Study 2 conducted confirmatory factor analyses (CFAs) using MPLUS 8.7 on the five core variables, including the mediator and moderator. The CFAs results are presented in Table 4. As shown in Table 4, the hypothesized five-factor model fit was significantly better than the other alternative models (χ^2^/*df* = 1.382, *RMSEA* = 0.045, *SRMR* = 0.054, *CFI* = 0.925, *TLI* = 0.918). It suggests that the five core variables of the present study demonstrated good discriminant validity and that the problem of common method bias was not severe, which allowed for the establishment of a five-factor model.

**Table 4:** Results of confirmatory factor analysis of Study 2.

**Models**	**χ^2^**	***df***	**χ^2^*/df***	***RMSEA***	***SRMR***	***CFI***	***TLI***
Five-factor model (IPV, MT, AI, TR, SL)	760.305	550	1.382	0.045	0.054	0.925	0.918
Four-factor model (IPV+MT, AI, TR, SL)	859.642	554	1.552	0.054	0.067	0.890	0.882
Three-factor model (IPV+MT, AI, TR+SL)	914.813	557	1.642	0.058	0.068	0.872	0.863
Two-factor model (IPV+MT+AI, TR+SL)	1151.770	559	2.060	0.075	0.099	0.787	0.774
One-factor model (IPV+MT+AI+TR+SL)	1314.432	560	2.347	0.085	0.080	0.729	0.712

N = 188. IPV, polychronicity; MT, multitasking; AI, AI-empowered task processing; TR, thriving at work; SL, self-leadership. +: factor added together.

#### 4.2.2 Descriptive statistics

Table 5 shows the means, standard deviations, correlation coefficients, and Cronbach's alpha for the core variables of Study 2. As shown in Table 5, there were all significant positive relationships between the variables. Cronbach's alpha for all variables were above of 0.75, and the scales all had high internal consistency.

**Table 5:** Mean, standard deviation, and correlations of all variables of Study 2.

**Variable**	**Mean**	**SD**	**1**	**2**	**3**	**4**	**5**	**6**	**7**	**8**	**9**
1. Gender	1.280	0.449									
2. Age	2.190	0.805	0.090								
3. Education	2.930	0.420	0.053	0.074							
4. Tenure	2.800	1.280	0.172^*^	0.505^**^	−0.028						
5. Polychronicity	4.823	0.944	0.134	0.216^**^	−0.063	0.074	(0.864)				
6. Multitasking	4.868	0.905	0.046	0.060	−0.051	0.041	0.855^**^	(0.893)			
7. AI-empowered task processing	5.169	0.800	−0.066	0.177^*^	−0.096	0.044	0.480^**^	0.271^**^	(0.748)		
8. Thriving at work	6.058	0.609	0.011	0.267^**^	−0.109	0.103	0.589^**^	0.340^**^	0.681^**^	(0.856)	
9. Self-leadership	5.050	0.645	0.027	0.265^**^	−0.061	0.056	0.699^**^	0.416^**^	0.831^**^	0.820^**^	(0.862)

N = 188. Gender: male = 1, female = 2. Age: 18–25 = 1, 26–30 = 2, 31–40 = 3, 41–50 = 4, above 50 = 5. Education: middle school = 1, senior high school = 2, bachelor degree = 3, master degree = 4, doctor degree and above = 5. Tenure: within 1 year = 1, 1–3 years = 2, 3–5 years = 3, 5–10 years = 4, 10 years and above = 5. Cronbach's alphas are shown in parentheses along the diagonal. SD, standard deviations. ^*^p < 0.05, ^**^p < 0.01.

#### 4.2.3 Common method bias analyses

Although Study 2 adopted a three-wave design to collect data, all five core variables were self-reported by employees, thereby multiple methods were used to examine the underlying severity of common method bias. (1) Harman's single-factor test. It showed that factors with eigenvalues >1 explained a total of 59.94% of the variance, and the first factor explained 32.61% of the variance, falling short of the threshold of 50% (Podsakoff et al., 2003). Thus, it is assumed that study 2 did not have a severe problem of common method bias. (2) Unmeasured Latent Method Construct (ULMC; Liang et al., 2007). The results showed that the model fit change after controlling for a latent variable was Δ*CFI* = 0.018, Δ*TFI* = 0.017, Δ*RMSEA* = 0.005, and Δ*SRMR* = 0.004, which were all < 0.02. Accordingly, common method bias of Study 2 was effectively controlled.

#### 4.2.4 Hypotheses testing

**Figure 4:**
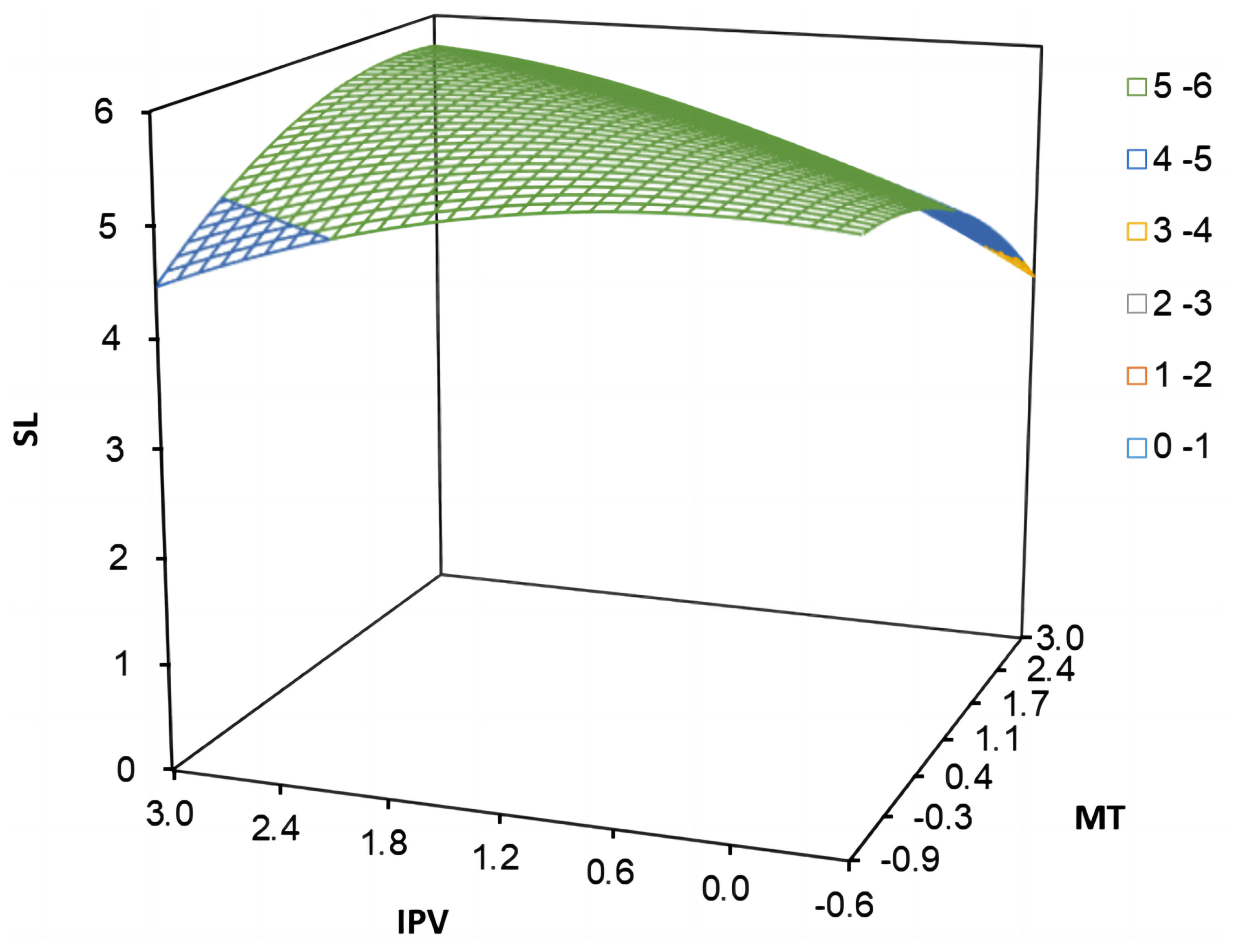
Response surface plot of polychronicity-multitasking fit influence employee self-leadership via thriving at work.

In Study 2, the proportion of “incongruent” samples was as high as 36.70%, far exceeding the 10% criterion, indicating the necessity of conducting the polynomial regression and response surface analysis. To test the mediating effect of thriving at work, we referred to Edwards and Cable (2009) and constructed a block variable for multitasking and polychronicity under the premise of not changing explanatory strength for the dependent variable. The bootstrapping analysis results of 10,000 resamples in Table 6 show that the mediating role of thriving at work between the relationship of polychronicity-multitasking fit and employee self-leadership (*indirect effect* = 0.377, *95% CI* = [0.335, 0.427], excluding zero). Thus, hypothesis 2 was supported. Moreover, from the visualized response surface plot with indirect effects in Figure 4, it could be seen that through the mediating effect of thriving at work, the values of employee self-leadership along the congruence line (*IPV* = *MT*) were significantly higher than those along the incongruence line (*IPV* = −*MT*). The “high-high” fit along the congruence line had a stronger positive effect on employee self-leadership through thriving at work than the case of “low-low” fit along the congruence line. Besides, “high-low” incongruent matching (see Figure 4, bottom left) had a stronger positive impact on employee self-leadership than ‘‘low-high” (see Figure 4, top right). Therefore, hypothesis 2 was further supported.

**Table 6:** Results of direct and indirect effects testing.

	**Thriving at work**	**Self-leadership**
Block variable	0.811^***^	0.623^***^
Thriving at work		0.465^***^
Indirect effects		0.377^***^
Indirect effects (95% *CI*)		[0.335, 0.427]

N = 188. Unstandardized coefficients were reported in the table. Control variables were not listed in the table. ^***^p < 0.001.

**Figure 5:**
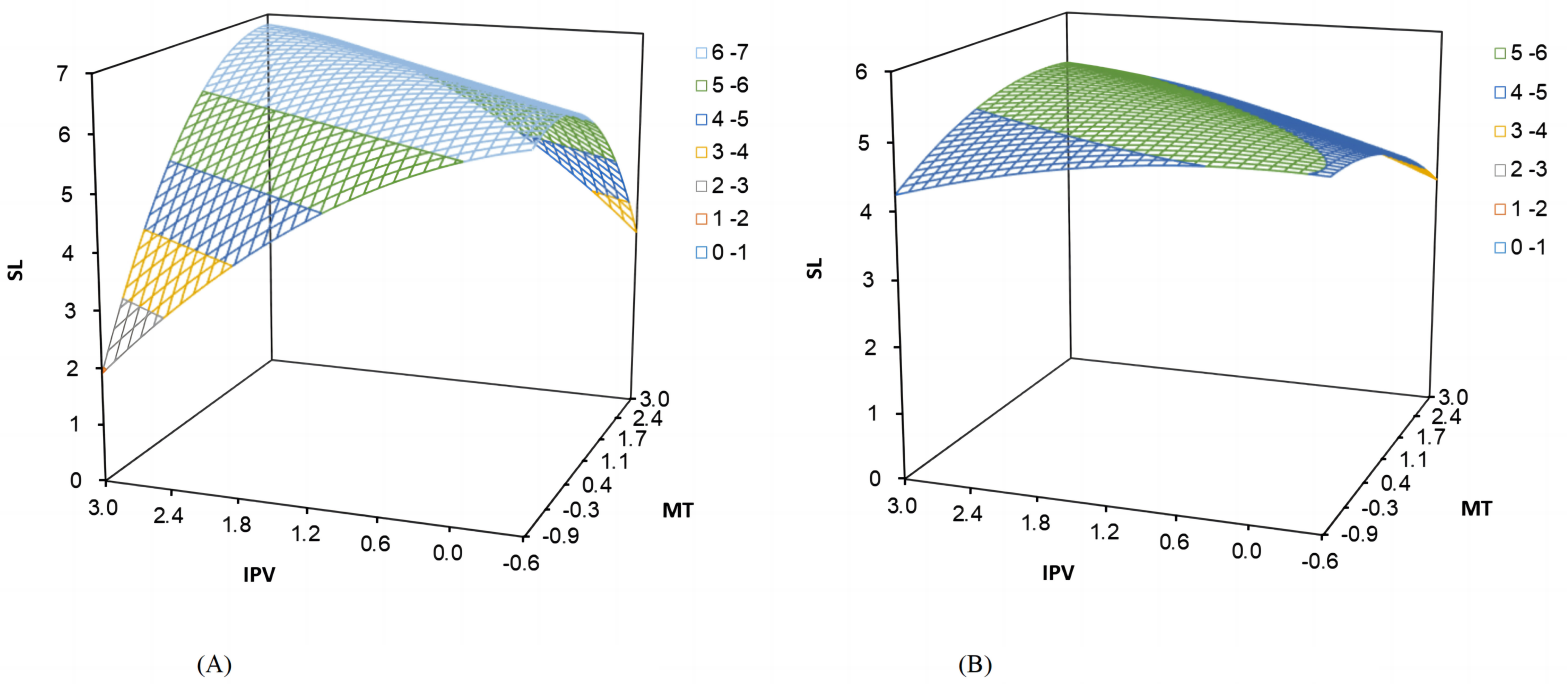
Response surface plots of the moderating role of AI-empowered task processing. **(A)** High AI-empowered task processing. **(B)** Low AI-empowered task processing.

For moderating testing, after adding the moderator and its five higher-order terms, i.e., *AI*×*IPV*, *AI*×*MT*, *AI*×*IPV*^2^, *AI*×*IPV*×*MT*, *AI*×*MT*^2^ to Model 7 in Table 7, the elevated model explanations Δ*R*^2^ was significant (Δ*R*^2^ = 0.035, *p* < 0.001), providing initial support for the moderating effect. Further, the mean value of the moderating variable AI-empowered task processing (*AI*) plus or minus one standard deviation (*SD*) were used to divide the high and low AI-empowered groups. The bootstrapping analyses of 10,000 resamples with 95% *CI* were employed to examine polynomial curvatures and slopes, respectively, for the different groups. The results were shown in Tables 7, 8 and response surface plots were displayed in Figures 5A, B).

**Table 7:** Results of polynomial regression analysis for moderating effects of Study 2.

	**Self-leadership**			
	**Model 4**	**Model 5**	**Model 6**	**Model 7**
***Intercepts***	3.377^***^	0.334	−0.072	4.689^***^
**Control variable**				
Gender	−0.166^*^	−0.090	0.016	0.018
Age	0.037	0.039	0.029	0.038^+^
Education	−0.019	−0.006	0.018	0.001
Tenure	−0.008	0.020	0.007	−0.005
**Polynomial variable**				
Polychronicity (*IPV*)	0.884^***^	1.937^***^	0.691^**^	0.213^***^
Multitasking (*MT*)	−0.490^***^	−0.327	0.246	−0.131^*^
*IPV*^2^		−0.591^***^	−0.419^***^	−0.046^**^
*IPV*×*MT*		0.929^***^	0.778^***^	0.062^*^
*MT*^2^		−0.460^***^	−0.428^***^	−0.035^+^
**Moderating variable**				
*AI*			0.427^***^	0.356^***^
*AI*×*IPV*				−0.072^**^
*AI*×*MT*				0.081^**^
*AI*×*IPV*^2^				−0.037^***^
*AI*×*IPV*×*MT*				0.100^***^
*AI*×*MT*^2^				−0.060^***^
*R*^2^	0.627	0.712	0.884	0.919
Δ*R*^2^	0.541^***^	0.084^***^	0.172^***^	0.035^***^
Δ*F*	131.334^***^	17.378^***^	262.933^***^	14.954^***^

N = 188.Δ*R*^2^ for model 4 refers to comparing with the baseline model merely included control variables. ^+^*p* < 0.10, ^*^p < 0.05, ^**^p < 0.01, ^***^p < 0.001.

**Table 8:** Results of response surface analyses for the moderating effect of AI-empowered task processing.

	**High AI group (Mean +SD)**	**Low AI group (Mean −SD)**
**Polynomial variable**		
Polychronicity (*IPV*)	0.024	0.178^***^
Multitasking (*MT*)	0.081^**^	−0.092^*^
*IPV*^2^	−0.144^***^	−0.065^***^
*IPV*×*MT*	0.324^***^	0.111^***^
*MT*^2^	−0.192^***^	−0.064^***^
**Congruence line (IPV** **=** **MT)**		
Slope (*b*_1_+*b*_2_)	0.104	0.086
95% *CI*	[0.089, 0.119]	[0.074, 0.098]
Curvature (*b*_3_+*b*_4_+*b*_5_)	−0.011	−0.018
95% *CI*	[−0.013, −0.009]	[−0.021, −0.015]
**Incongruence line (IPV** **=** **–MT)**		
Slope (*b*_1_−*b*_2_)	−0.057	0.269
95% *CI*	[−0.065, −0.049]	[0.231, 0.308]
Curvature (*b*_3_−*b*_4_+*b*_5_)	−0.659	−0.240
95% *CI*	[−0.753, −0.565]	[−0.274, −0.206]
Side-shift (*b*_2_−*b*_1_)/[2 × (*b*_3_−*b*_4_+*b*_5_)]	−0.043	0.561
95% *CI*	[−0.049, −0.037]	[0.481,0.641]

N = 188. ^*^p < 0.05, ^**^p < 0.01, ^***^p < 0.001.

The relationship between polychronicity-multitasking fit and employee self-leadership under high levels of AI empowerment still existed, with significantly negative response surface curvature along the incongruence line (*IPV* = −*MT*) (*b*_3_−*b*_4_+*b*_5_ = −0.659, 95*% CI* = [−0.753, −0.565]). The 95% *CI* of the first principal axis slope, *p*_11_, was [0.860, 1.510], including 1. This suggests high-level AI empowered task processing strengthened the positive impact of congruent matching of polychronicity and multitasking on employee self-leadership compared to incongruent states. Similarly, under low-level AI empowerment, congruent matching predicted higher self-leadership than incongruent matching (*b*_3_−*b*_4_+*b*_5_ = −0.240, 95*% CI* = [−0.274, −0.206]). However, according to the magnitude of curvature along the incongruence line for the high and low AI-empowered task processing groups and considering the degree of “inverted U-shaped” curvature along the incongruence line presented in the response surface plot in Figures 5A, B, the curvature was significantly steeper in the high AI condition and showed a more pronounced concave shape. According to the visualized plots, compared with the low AI empowerment, the employee self-leadership in the high AI condition valued higher in the congruent matching. Specifically, the highest point of the congruent matching in Figure 5A was close to 7 and in Figure 5B was around 5. In contrast, the incongruent matching states took a lower value. The lowest point of the incongruent matching in Figure 5A was around 2, while in Figure 5B was around 4. In summary, employee self-leadership was lower at high levels of AI-empowered task processing under incongruent matching and was higher at high AI empowerment under congruent matching. Thus, hypothesis 3a was supported.

In both cases of incongruent fit, when there was a high level of AI-empowered task processing, the values of employee self-leadership were about 2 and 3 for “high-low” and “low-high” state, respectively. In contrast, in the low AI empowerment group, the values were significantly higher than 3.5. Hypothesis 3b was partially supported. Further, as shown in Table 8, the slope of the response surface along the incongruence line (*IPV* = −*MT*) in the high AI empowerment group was significantly negative (*b*_1_−*b*_2_ = −0.057, 95*% CI* = [−0.065, −0.049]). The side-shift along the incongruence line (*IPV* = −*MT*), as proposed by Edwards and Parry (1993), was also significantly < 0 {(*b*_2_−*b*_1_)/[2 × (*b*_3_−*b*_4_+*b*_5_)] = −0.043,95*% CI* = [−0.049, −0.037]}. This suggests that, compared to the case of “high-low” incongruent fit, the “low-high” state might predict higher employee self-leadership. However, in the low AI empowerment group, the slope of the response surface along the incongruence line (*IPV* = −*MT*) was significantly positive (*b*_1_−*b*_2_ = 0.269, 95*% CI* = [0.231, 0.308]). The side-shift along the incongruence line (*IPV* = −*MT*) was also significantly >0 {(*b*_2_−*b*_1_)/[2 × (*b*_3_−*b*_4_+*b*_5_)] = 0.561,95*% CI* = [0.481. 0.641]}. It indicates that “high-low” state had a stronger positive impact on employee self-leadership than “low-high” state. To sum up, the slope along the incongruence line changed from positive to negative as the AI empowerment level increased. That is, for employee self-leadership, as the level of AI-empowered task processing increased, the state of “high-low” state being more potent than “low-high” state would change to the state of “low-high” state exceeding “high-high” state. Thus, hypothesis 3b was supported.

In addition, according to the response surface plots, it could be drawn that the height of the surface along the incongruence line (*IPV* = −*MT*) in the response surface of the low AI group in Figure 5B was significantly higher in the “high-low” region of “IPV > MT” than in the “low-high” region of “MT > IPV” and the lowest value of employee self-leadership occurred in the “low-high” region. It indicates that the positive impact of the “high-low” state on employee self-leadership was stronger than that of the “low-high” state. The height of the surface along the incongruence line (*IPV* = −*MT*) in the response surface of the high AI empowerment group in Figure 5A was significantly lower in the “high-low” region of “IPV > MT” than in the “low-high” region of “MT > IPV” and the lowest value of employee self-leadership occurred in the “high-low” region. It demonstrates that employee self-leadership was higher in the “low-high” state compared to the “high-low” state, which further supported the hypothesis 3b.

## 5 Discussion

To elucidate how the prevailing multitasking practices among employees in organizations can synergistically influence their self-leadership within the context of artificial intelligence empowerment, this study conducts two time-lagged survey studies to examine whether, how, and when polychronicity-multitasking fit promotes employees' self-leadership. We found that the congruence in polychronicity-multitasking fit predicted higher levels of self-leadership compared to incongruence. And the more congruent, the greater the employees' self-leadership. Within the two states of the incongruence, the “high-low” state stimulated self-leadership better than the “low-high” state. We also found that thriving at work mediated the impact of polychronicity-multitasking fit on self-leadership. Moreover, AI-empowered task processing enhanced the positive relationship between the congruence in polychronicity-multitasking fit and employee self-leadership, while mitigating the impact of incongruence in polychronicity-multitasking fit on self-leadership. With the influence of AI empowerment, employee self-leadership became higher in the “low-high” state than in the “high-low” state.

### 5.1 Theoretical implications

This study has made three focal contributions. First, our findings enhance the existing literature on JD-R theory by elucidating the distinct impact on self-leadership resulting from diverse matching states of three multitasking-related elements (i.e., fit between polychronicity, multitasking and AI-empowered task processing) in the era of artificial intelligence (Zhang and Parker, 2019). We reveal that the development of employee self-leadership is primarily contingent upon the fit of multidimensional states rather than unidimensional multitasking orientations or requirements (Stewart et al., 2019). This sheds light on potential factors contributing to the significant disparities observed in previous research regarding the effects of polychronicity and multitasking (Howard and Cogswell, 2022; Mattarelli et al., 2015). In this paper, we present the four fundamental states of polychronicity-multitasking fit, based on JD-R theory, which vividly exemplify the dual pathways of work resources (i.e., polychronicity) and demands (i.e., multitasking), as well as the influence of “buffering” and “coping” effects on employee self-leadership. This aligns with Howard and Cogswell (2022) call to investigate the varying effects of excessive and deficient multitasking pressures on employee work motivation. Moreover, our analysis reveals that the role of AI empowerment as either a “resource” serving as a “co-pilot” in task processing, or a “demand” imposing additional burden or exacerbating imbalances for employees necessitates specific investigations tailored to different states of polychronicity-multitasking fit. This has further enriched the theoretical insights of JD-R theory from the vision of burgeoning artificial intelligence technology at work.

Second, by innovatively introducing the FITT framework into the field of organizational management, this study extents the work-related frontier technology dimension in the work environment beyond the classic “person-job fit” framework (Chowdhury et al., 2023; Makarius et al., 2020), which potently resonates with Howard and Cogswell (2022) calling for directly integrating P-E fit theory into the polychronicity research. This study comprehensively illuminates the diverse impacts of different polychronicity-multitasking fit on employee self-leadership within varying degrees of AI-empowered task processing, with a particular focus on the disruptive influences of AI technologies on employees' self-growth at work (Vrontis et al., 2022). The findings of this study indicate that high levels of AI-empowered task processing will positively influence self-leadership of employees who are of congruence in polychronicity-multitasking fit, while negatively affecting those who are in incongruent matching. In particular, when all three elements in FITT framework are maintained at a high level (i.e., the “high-high-high” state), employees can optimize both internal and external resources at workplace. However, high AI empowerment within the organization may exacerbate the imbalanced working state of employees in the “high-low” state arising from weak external incentives, thus its inhibitory impact on self-leadership development will reach its peak in a “high-low-high” state. We enrich the existing body of literature by demonstrating the nuanced impact of AI empowerment on employees in diverse work states, aiming to determine the optimal condition for harnessing AI technology to foster employee self-leadership. In this regard, this study calls attention to zooming in the intricate and contingent value and challenges of artificial intelligence for employee self-development.

Third, this study contributes to the self-leadership literature by exploring the linkage between polychronicity-multitasking fit and self-leadership, and by identifying the role of AI and its influencing mechanism. Prior studies have primarily concentrated on exploring how individual traits and leadership styles shape employees' self-leadership, with limited focus on the interactive view of employees' working state and working environment (Harari et al., 2021; Tang et al., 2023). Grounded on the FITT framework, the present study unfolds that different fit states of polychronicity, multitasking, and AI-empowered task processing would result in varying levels of self-leadership. Besides, by identifying the mediating role of thriving at work in the focal relationship, we also extents the research on the socially embedded model of thriving at work and broadens the exploration of the motivational process of self-leadership development (Goh et al., 2022; Harari et al., 2021). The present paper presents a groundbreaking and multi-faceted viewpoint on cultivating individual self-leadership within organizations empowered by emerging artificial intelligence, marking the inception of a new research stream that delves into the dynamic fit perspective of self-leadership development.

### 5.2 Practical implications

The present study provides practical guidance for the enhancement of human resource management system and the improvement of organizational human resources quality in response to the advent of artificial intelligence in the workplace.

First, it is important to emphasize employees' balanced state of supply and demand for multitasking in the context of artificial intelligence. To achieve the optimal facilitating effect of “1+1 > 2,” employees with significant disparity in polychronicity-multitasking fit should be guided to make positive adjustments toward the direction of congruent matching in terms of individual preference and work demand (Howard and Cogswell, 2022). During this process, the intrinsic motivation adjustment of employees assumes paramount significance. Thus, in an incongruent state, employees should be given sufficient autonomy to adapt their multi-task pressures in accordance with their preferences to rectify the mismatched state and further foster self-leadership (Mueller and Niessen, 2019).

Second, there is a necessity to pay attention to the ambivalent impact of task-aided AI technologies on both employees and organizations. Managers should exercise caution when implementing and advancing artificial intelligence, avoiding the adoption of standardized approaches for deploying intricate AI within different organizational working contexts. Instead, employees should be provided with flexible and autonomous opportunities for learning and applying artificial intelligence according to their specific work preferences, task processing status, and level of individual autonomy, rather than being subjected to rigid technological threshold requirements (Cheng et al., 2023). On the one hand, managers should strive to create a conducive environment for employees, characterized by enhanced artificial intelligence support and enriched opportunities for technology learning. On the other hand, managers need to be especially vigilant about the significant impediment of AI to the growth of employees under the state of mismatch.

Third, the cultivation of individual positive working states and the facilitation of autonomous factors in employee self-leadership development necessitate meticulous attention. The presence of individual autonomy as a positive internal resource can significantly compensate for the work depletion caused by intense external demands (Nahrgang et al., 2011). Managers should prioritize the empowerment of employees, enabling them to work autonomously. This can be achieved by proactively providing them with favorable work states that allow for the exertion of their autonomy and motivation, meanwhile guiding them toward maintaining a high level of commitment and progress in their job orientation.

### 5.3 Limitations and future research

This paper has several limitations, which present some promising directions for future research. First, despite the multi-wave design adopted, the measures in two studies were self-rated, thus complete elimination of potential common method bias cannot be guaranteed (Podsakoff et al., 2012). Future studies may consider adopting other data collection sources and analysis methods to explore how fit between polychronicity and multitasking could influence employee self-leadership.

Second, the limited sample size and study duration may prevent strict causal inferences from being drawn from the results. To address the possibility of reverse causality, future research could adopt laboratory experiments, field experiments, or longitudinal studies to better clarify the causal relationship between polychronicity-multitasking fit and employee self-leadership.

Third, this study reveals the mediating role of thriving at work between polychronicity-multitasking fit and self-leadership. Future research could further expand the influencing mechanism from other theoretical perspectives. Besides, future study could enrich the antecedents of self-leadership from other more intricate aspects, such as the “Fit between Individuals, Tasks, Technology and Environment” (FITTE) framework that incorporates the environmental dimension in the artificial intelligence era.

## Data Availability

The original contributions presented in the study are included in the article/supplementary material, further inquiries can be directed to the corresponding author/s.
